# Tetramethylpyrazine enhanced the therapeutic effects of human umbilical cord mesenchymal stem cells in experimental autoimmune encephalomyelitis mice through Nrf2/HO-1 signaling pathway

**DOI:** 10.1186/s13287-020-01700-z

**Published:** 2020-05-19

**Authors:** Lianshuang Zhang, Xifeng Wang, Xueyan Lu, Yanchao Ma, Xin Xin, Xiaomin Xu, Siyuan Wang, Yun Hou

**Affiliations:** 1 Department of Histology and Embryology, College of Basic Medicine, Binzhou Medical University, Yantai, China; 2 Department of Critical Care Medicine, Yu Huang Ding Hospital, Qingdao University, Yantai, China; 3 Department of Immunology, College of Basic Medicine, Binzhou Medical University, Yantai, China

**Keywords:** Mesenchymal stem cells, Tetramethylpyrazine, Oxidative stress, Nrf2/HO-1 signaling pathway, Experimental autoimmune encephalomyelitis, Multiple sclerosis

## Abstract

**Introduction:**

The therapeutic effects of mesenchymal stem cells (MSCs) have been limited by their apoptosis induced by oxidative stress after delivery into the injured sites. Therefore, strategies designed to improve the MSC therapeutic efficacy need to be explored. Tetramethylpyrazine (TMP) can promote the proliferation and differentiation of neural stem cells. In this study, we first evaluated the effects and mechanism of TMP on H_2_O_2_-stimulated human umbilical cord MSCs (hUCMSCs) and then further investigated the therapeutic effects of TMP-stimulated hUCMSCs on experimental autoimmune encephalomyelitis (EAE) mice.

**Methods:**

The toxicity of hUCMSCs against of TMP was determined by cell count kit-8 (CCK-8) assay. The effects of TMP on the hUCMSC cell cycle, the reactive oxygen species (ROS) production, and the apoptosis of H_2_O_2_-stimulated hUCMSCs were determined by flow cytometry. The expression of malondialdehyde (MDA) and superoxide dismutase (SOD) were also measured by colorimetry. The signaling pathway of TMP induced on H_2_O_2_-stimulated hUCMSCs was investigated by western blot. EAE was induced using immunization with MOG35-55 in C57BL/6 mice. The inflammatory cell infiltration and demyelination were detected by immunofluorescence staining. The blood-brain barrier (BBB) disruption was detected by Evans blue (EB) stain and the expression of tight junction protein (ZO-1) by western blot.

**Results:**

TMP significantly increased cell viability and changed the cell cycle of hUCMSCs. In addition, TMP (100 μM) significantly reduced intracellular ROS production, expression of MDA, and apoptosis, but increased expression of SOD through nuclear factor-erythroid 2-related factor-2 (Nrf2)/heme oxygenase 1 (HO-1) signaling pathway in H_2_O_2_-stimulated hUCMSCs. Most importantly, compared with wild hUCMSCs, TMP-stimulated hUCMSCs significantly ameliorated EAE, by attenuation of inflammation, demyelination, and BBB disruption.

**Conclusion:**

The TMP-stimulated hUCMSCs provide a potential therapeutical protocol to enhance the therapeutic effects of hUCMSCs in multiple sclerosis.

## Introduction

Human umbilical cord mesenchymal stem cells (hUCMSCs) have many advantages including non-invasive collection procedure, low risk of infection, low immunogenicity, and multi-potency [1]. These advantages make hUCMSCs widely applied to the transplantation therapy of diseases, including cerebral ischemia-reperfusion injury and ischemia/reperfusion-induced acute kidney injury [2, 3]. However, the apoptosis of MSCs induced by oxidative stress, a major element with negative influence on transplanted MSCs, affects the transplantation efficiency of MSCs after delivery into the injured sites [4]. It was demonstrated that the apoptosis of MSCs was associated with significant increasement of ROS generation [5, 6]. Therefore, the low-survival rate of transplanted hUCMSCs induced by oxidative stress needs to be solved urgently.

Pharmacological pretreatment has been shown to be a rational approach to reinforce the MSCs to withstand the ischemic and reperfusion injury environment [7]. Tetramethylpyrazine (TMP), an alkaloid monomer extracted from the traditional Chinese herb *chuan xiong*, has been shown having the effects of anti-inflammatory, free radical scavenging, and anti-apoptosis and has been used in the treatment of cardiovascular and cerebrovascular diseases in clinical treatment. Recent studies showed that TMP can inhibit apoptosis in various cells, such as hypoxia-induced myocardial cell apoptosis [8] and hydrogen peroxide-induced oxidative damage in human umbilical vein endothelial cells [9]. Nuclear factor-erythroid 2-related factor-2 (Nrf2)/heme oxygenase 1 (HO-1) is one of the most important defensive signaling pathways for regulating the activity of antioxidants. Studies have shown that activating the Nrf2/HO-1 signaling axis reduces oxidative stress through antioxidant, anti-inflammatory, reducing mitochondrial damage, regulating intracellular calcium flow, and regulating apoptosis, pyroptosis, ferroptosis, and autophagy [10]. Although many studies have proved that various cells can be protected against oxidative stress and apoptosis, there are no relevant studies on cytoprotective effects of TMP on hUCMSCs by activating Nrf2/HO-1 pathway. Therefore, the first aim of our study was to explore whether TMP alleviates oxidative stress injury of hUCMSCs by activating Nrf2/HO-1 pathway.

Multiple sclerosis (MS) is an inflammatory and demyelinated disease of the central nervous system (CNS), which is characterized by inflammation, BBB disruption, and demyelination, and its rational animal model is experimental autoimmune encephalomyelitis (EAE) [11]. MSCs have exhibited therapeutic effects on EAE mice to reduce inflammation and protect the myelination [11–13]. Therefore, the second purpose of this study is that we planned to figure out whether the therapeutic effects of TMP-hUCMSCs were enhanced to remittance severity of EAE.

In this study, we investigated whether TMP alleviates oxidative stress injury of hUCMSCs by activating Nrf2/HO-1 pathway and the therapeutic effects of transplanted TMP-hUCMSCs in EAE mice. We find that TMP significantly increased cell viability and changed the cell cycle of hUCMSCs. Moreover, TMP (100 μM) significantly reduced intracellular ROS production, expression of MDA, and apoptosis, but increased expression of SOD through Nrf2/HO-1 signaling pathway in H_2_O_2_-stimulated hUCMSCs. Most importantly, compared with wild hUCMSCs, TMP-stimulated hUCMSCs significantly ameliorated EAE, by attenuation of inflammation, demyelination, and BBB disruption. Therefore, the TMP-stimulated hUCMSCs provide a potential therapeutical protocol to enhance the therapeutic effects of hUCMSCs in inflammatory diseases of CNS, such as MS, stroke, and spinal cord injury.

## Materials and methods

### Isolation and characterization of hUCMSCs

The sterile umbilical cords were retrieved from the Yantai affiliated hospital of Binzhou Medical University after informed consent of pregnant women and were approved by the Institutional Ethics Committee. The details of isolation and characterization of hUCMSCs refer to our previous study [14].

### Determination of cell viability by cell counting kit-8 (CCK-8) assay

The viability of hUCMSCs was determined using a CCK-8 kit according to the manufacturer’s protocol (Beyotime, Shanghai, China). MSCs cultured in 96-well microplates were respectively treated with TMP (Sigma-Aldrich, MO, USA) at concentrations of 0, 1, 25, 50, 100, 150, and 200 μM for 24 h. Then, a volume of 10 μL CCK-8 solution was added to each well for 40 min and the microplates were measured at 490 nm using a microplate reader. According to this result, we chose TMP (100 μM) in the following experiments.

### Determination of hUCMSC characterization to TMP

hUCMSCs treated with or without TMP were collected to identify the cells by detecting the expression of the MSC surface markers stained with IgG1, IgG2b, CD44, CD73, CD90, CD34, CD45, and HLA-DR antibodies (all purchased from BD Bioscience, Franklin Lakes, NJ, USA) through flow cytometry. Then, the cells were cultured and dyed with osteogenesis or lipogenesis kit (Weitong Biosciences, Shenzhen, China) according to the manufacturer’s introduction to characterize the differentiation capacity of the hUCMSCs.

### Determination of cell cycle analysis by flow cytometry

hUCMSCs cultured in 6-well plates were respectively treated with TMP at concentrations of 50, 100, and 150 μM for 24 h. The cells were collected, fixed, and added with 100 μL RNaseA for 30 min at 37 °C. After 400 μL PI was added and mixed uniformly, the cells were dyed in dark for 30 min at 4 °C. Then, the cells were detected by flow cytometry. We analyzed the proportion of each phase cells with MultiCycle V3.0 software. For example, cells in G0–G1 phase with the least DNA content did not start DNA replication, which is shown as the first peak. S phase was the process in which the DNA copied from one time to two times, which was shown as the second low but wide peak. And G2–M phase was the cells with two times DNA, which was shown as the third peak. By measuring the proportion of cells in each phase of the cell cycle, we can get the degree of DNA replication and division of each group of cells.

### Measurement of intracellular ROS generation by flow cytometry

The ROS generation was measured through fluorescence intensity of dichlorofluorescein (DCF) by flow cytometry. Briefly, hUCMSCs cultured in 6-well plates were respectively pretreated with TMP (100 μM) for 24 h following with or without *N*-acetyl-l-cysteine (NAC) (1 μM) (Sigma) for 4 h. Then, cells were washed and exposed to H_2_O_2_ (500 μM) for 2 h [14]. The cells were collected, digested, and resuspended with 200 μL 2,7-dichlorofluorescein-diacetate (DCFH-DA) (Nanjingjiancheng, China) of 1 μM/L for 15 min at room temperature in the dark, and then, it was centrifuged and resuspended with 400 μL PBS for flow cytometry.

### Measurement of malondialdehyde (MDA) and superoxide dismutase (SOD)

hUCMSCs cultured in 6-well plates were respectively pretreated with TMP (50, 100 μM) for 24 h following with or without NAC (1 μM) for 4 h. Then, cells were washed and exposed to H_2_O_2_ (500 μM) for 2 h. The protein was extracted by a protein extraction kit (Jiancheng Bioengineering, Nanjing, China) and quantified by a BCA protein kit (Solarbio Life Sciences, Beijing, China).

The concentration of MDA was determined using an MDA kit (Jiancheng Bioengineering). The quantified protein of cells together with mixtures provided in the MDA kit were kept in a boiling water bath for 1 h; then, the mixture was centrifuged and the supernatants were measured at 532 nm with a microplate reader (Molecular Devices, MA, USA).

The concentration of SOD was determined using an SOD kit (Jiancheng Bioengineering). The quantified protein of cells together with mixtures provided in the SOD kit were kept at 37 °C for 20 min; then, the total mixtures were measured at 450 nm with microplate reader (Molecular Devices).

### Determination of cell apoptosis by flow cytometry

The apoptosis was determined with both FITC-conjugated Annexin V and propidium iodide (PI) apoptosis detection kit (Beyotime). hUCMSCs cultured in 6-well plates were respectively pretreated with TMP (100 μM) for 24 h following with or without NAC (1 μM) for 4 h. Then, cells were washed and exposed to H_2_O_2_ (500 μM) for 2 h [15]. The cells were collected, resuspended, and incubated with 500 μL binding buffer mixing with 5 μL Annexin V-FITC and 5 μL PI in the dark for 20 min for flow cytometry. The different cell populations were identified by the different labeling patterns in the Annexin V-PI analysis. FITC−/PI− were designated as live cells; FITC−/PI+ were designated as cell fragments; FITC+/PI− were designated as early apoptotic cells; FITC+/PI+ were designated as late apoptotic cells; the sum of the early apoptosis rate and the late apoptosis rate was finally calculated as the total rate of apoptosis.

### Nrf-2/HO-1 signaling pathway protein detection by western blot

hUCMSCs cultured in 6-well plates were respectively pretreated with TMP (50, 100 μM) for 24 h following with or without ML385 (1 μM) for 4 h. ML385 is an inhibitor of Nrf-2. Then, cells were washed and exposed to H_2_O_2_ (500 μM) for 2 h. The protein was extracted by a protein extraction kit (Jiancheng Bioengineering) and quantified by a BCA protein kit (Solarbio Life Sciences). Protein samples (30 μg) were loaded and electrophoresed on 10% SDS-polyacrylamide gels, electrotransferred to polyvinylidene fluoride membranes (Millipore, Billerica, MA, USA), blocked with 5% skim milk for 2 h, and incubated with the primary antibodies: Nrf-2 (1:500), HO-1 (1:500), and β-actin (1:1000) (all purchased from Cell Signaling) at 4 °C overnight. The transmembranes were then washed and incubated with a secondary antibody (1:5000, Bioss, Beijing, China) for 1 h to visualize the bands using an ECL detection kit (Meilun Biotechnology, Dalian, China). The densitometry was quantified using the Image Lab Software (Bio-Rad Laboratories Inc., Hercules, CA, USA).

### Induction of EAE and treatments

The animal experiments were performed in line with the Guide for the Care and Use of Laboratory Animals by the National Institutes of Health and the Ethical Committee of Binzhou Medical University. EAE was established in C57BL/6 mice (female, 10 weeks old, Pengyue, Jinan, China) by immunization with MOG35-55 (Hooke Laboratories, Lawrence, USA) according to the manufacturer’s instructions, and the details refer to our previous study [16]. The clinical scores were recorded daily from day 8 post-immunization according to Kono’s 5 score and Hooper’s 7 score [16, 17]. Mice were randomly divided into three groups: phosphate-buffered saline (PBS) (*n* = 20), hUCMSCs (*n* = 20), and TMP-stimulated hUCMSCs (*n* = 20). All treatments were carried out on day 13 after immunization. The hUCMSCs and TMP-hUCMSCs (both 1 × 10^6^/mouse) were injected by tail. The EAE mice were sacrificed on day 42 after immunization for further experiments. Hematoxylin-eosin (HE) staining and Luxol fast blue (LFB) were routinely performed.

### Immunofluorescence staining

The EAE mice were sacrificed, and frozen lumbar spinal cords were obtained for immunofluorescence staining. The lumbar spinal cord sections were incubated at 4 °C overnight with primary antibodies: NLRP3 (1:2000) and myelin basic protein (MBP) (1:300) (both were purchased from Cell Signaling). Then, the sections were washed and incubated the alexa-488 (Sigma-Aldrich, St. Louis, SA) for 2 h for the detection of fluorescence signals using the laser scanning confocal microscopy (Carl Zeiss, Oberkochen, Germany).

### BBB disruption determination by extravasation of Evans blue (EB)

For the details in regard to the EB dye, please refer to our previous study [18]. The EB solution was injected 4 h before the mice were sacrificed.

### Tight junction proteins of lumbar spinal cord detection by western blots

Fresh spinal cords were obtained and homogenized for gel electrophoresis. The transmembranes were incubated at 4 °C overnight with primary antibodies: β-actin (1:1000) (Cell signaling) and ZO-1 (1:200) (NOVUS Biologicals, CO, USA) followed by horseradish peroxidase-conjugated secondary antibodies (Cell Signaling). To detect the immunoreactive bands, visualize the bands using an ECL detection kit (Meilun Biotechnology, Dalian, China). The densitometry was quantified using the Image Lab Software (Bio-Rad Laboratories Inc., Hercules, CA, USA).

### Statistical analysis

All of the data were analyzed using SPSS 23.0. Differences among all groups were analyzed by one-way analysis of variance (ANOVA) followed by post hoc Bonferroni corrections. Values are reported as mean ± standard error (SE). *P* < 0.05 was considered to indicate a statistically significant difference.

## Result

### TMP promoted the viability of hUCMSCs and changed the cell cycle of hUCMSCs

**Fig. 1:**
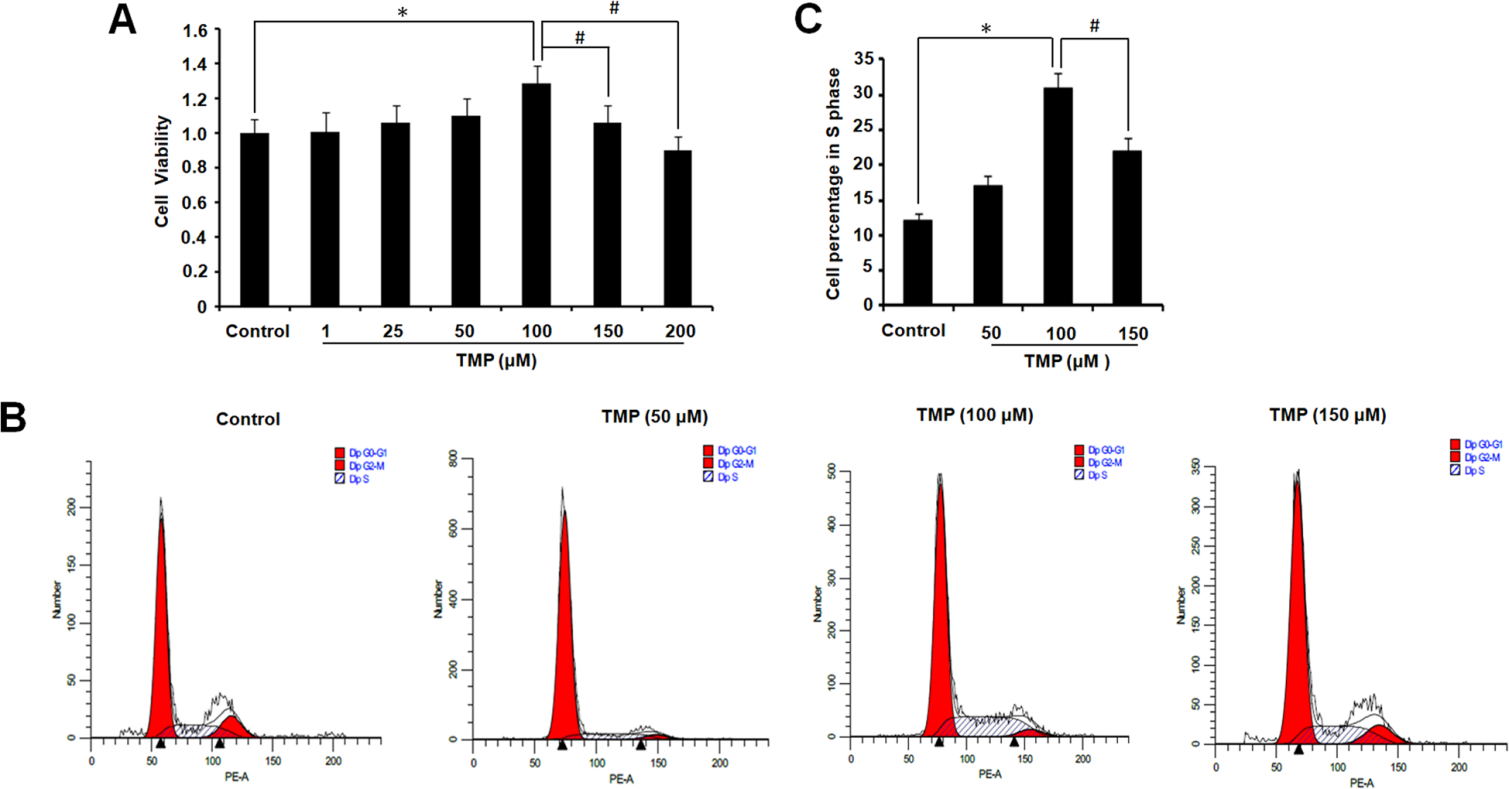
TMP changes the cell cycle of hUCMSCs and improves the cellular viability. **a** hUCMSC viabilities were analyzed by CCK-8 assay. **b** The cell cycle was determined by flow cytometry. **c** Statistical analysis of the number of cells in each phase in each group. Columns, mean; bars, SEM. The asterisk indicates significant difference compared to the control group (*P* < 0.05); the number sign indicates significant difference compared to the TMP (100 μM) group. The results are representative of three independent experiments

hUCMSCs were treated with TMP at different concentrations (1, 25, 50, 100, 150, and 200 μM) to the detect the effect of TMP on the hUCMSC viability, respectively. The results indicated that the TMP dose dependently increased hUCMSC viability, and hUCMSC viability was significantly increased in the presence of TMP (100 μM) (*P* < 0.05), but no significant changes with TMP (1, 25, 50 μM). The viability of hUCMSCs was decreased in the presence of TMP at concentration of 150 μM and 200 μM (Fig. 1a).

The cell cycle distribution was assessed by monitoring the intensity of propidium iodide fluorescence. As shown in Fig. 1b, c, the cell division was figured out by measuring the percentage of cells accumulated in the S phase. hUCMSCs treated with TMP (100 μM) showed a significantly higher distribution in the S phase compared with that in the control group (*P* < 0.05), but no significant changes in the TMP (50 μM) group. Also, TMP (150 μM) decreased the percentage of hUCMSC distribution in the S phase. These results indicate that TMP (100 μM) could promote the cell viability and differentiation of hUCMSCs thus promoting their proliferation.

### The effects of TMP on hUCMSC phenotype and differentiation capability

**Fig. 2:**
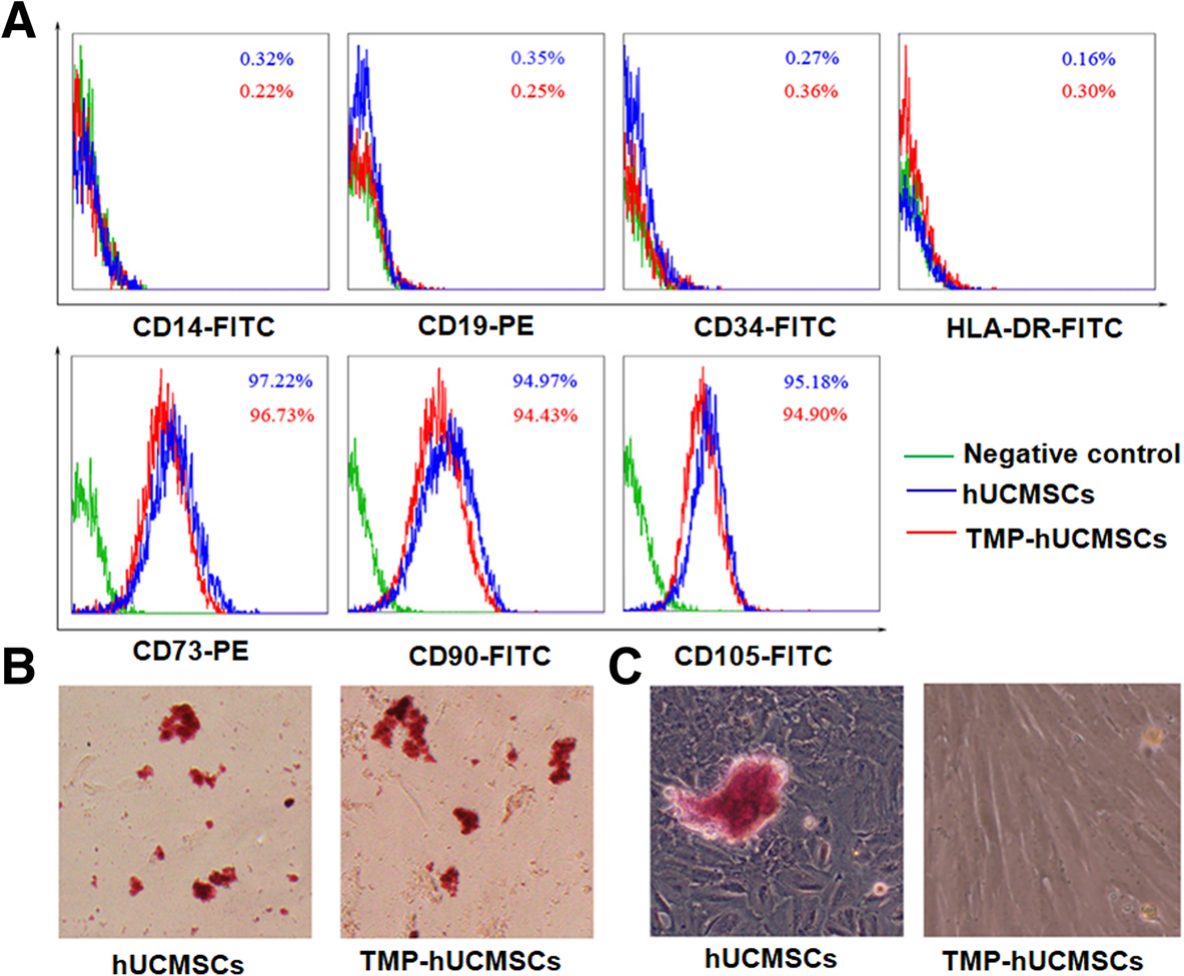
Effects of TMP on hUCMSC characterization. **a** FACS analysis of the effect of TMP on hUCMSC phenotype. Wild-type (blue lines) and TMP-treated hUCMSCs (red lines) were labeled with antibodies for MSC phenotypic surface markers. The negative controls stained with IgG1 and IgG2b are shown in green lines. **b** The effects of TMP on the differentiation capability of hUCMSCs. The results are representative of three independent experiments

The effects of TMP on hUCMSC phenotype were detected by flow cytometry to determine their surface markers. Similar to wild-type hBM-MSCs, TMP-treated hUCMSCs were strongly positive for CD73, CD44, and CD90 and negative for CD34, CD45, and HLA-DR (Fig. 1b). There were no significant differences in the number of positive stained surface markers between wild-type hUCMSCs and TMP-stimulated hUCMSCs (Fig. 2a). We also detected the effects of TMP on hUCMSC differentiation capacity; the results showed that TMP did not change the hUCMSC differentiation capacity to osteocytes stained by Alizarin Red S or adipocytes stained by Oil Red O, respectively (Fig. 2b). Taken together, these results suggest that TMP could not affect the characterization of hUCMSCs.

### TMP alleviated H_2_O_2_-induced oxidative stress

**Fig. 3:**
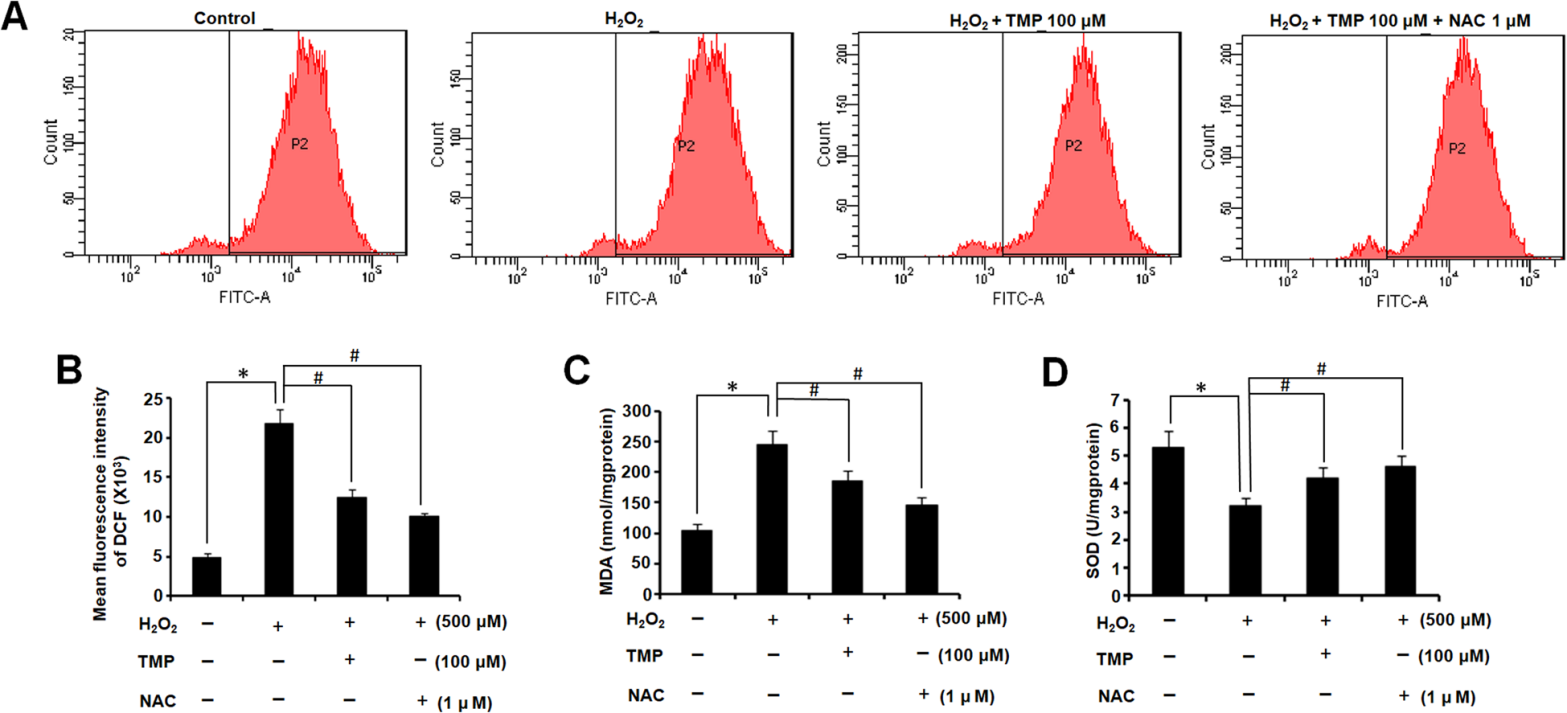
TMP alleviates H_2_O_2_-induced oxidative stress. **a** The fluorescence intensity of ROS in each group was measured by flow cytometry. **b** Statistical analysis of the mean fluorescence intensity of ROS in each group. **c** Statistical analysis of the mean concentration of MDA in each group. **d** Statistical analysis of the mean concentration of SOD in each group. Columns, mean; bars, SEM. *P < 0.05 compared to the control group; ^#^*P* < 0.05 compared to the H_2_O_2_-treated group. The results are representative of three independent experiments

hUCMSCs treated with H_2_O_2_ were used as the cell model of oxidative stress. NAC is used as a positive control, as it is a glutathione precursor with potent antioxidant and anti-inflammatory properties [19]. The mean fluorescence intensity of DCF in each group was measured by flow cytometry. As shown in Fig. 3a, b, the mean fluorescence intensity of hUCMSCs induced by H_2_O_2_ (500 μM) significantly increased, compared with the control group (*P* < 0.05). In contrast, pretreatment with TMP (100 μM) or NAC before exposure to H_2_O_2_ obviously decreased the fluorescence intensity of DCF, compared to the group treated with H_2_O_2_ alone (*P* < 0.05). In order to comprehensively illustrate the effects of TMP on the oxidative stress, we also measured the concentration of MDA and SOD. MDA is a marker for peroxidation. The expression of MDA showed a similar expression pattern with ROS that TMP or NAC pretreatment significantly decreased the expression of MDA, compared to the group treated with H_2_O_2_ alone (*P* < 0.05). SOD is a marker for antioxidation, and its expression showed an opposite expression pattern with ROS that TMP or NAC pretreatment significantly increased the expression of SOD, compared to the group treated with H_2_O_2_ alone (*P* < 0.05).

### TMP alleviated H_2_O_2_-induced apoptosis of hUCMSCs

**Fig. 4:**
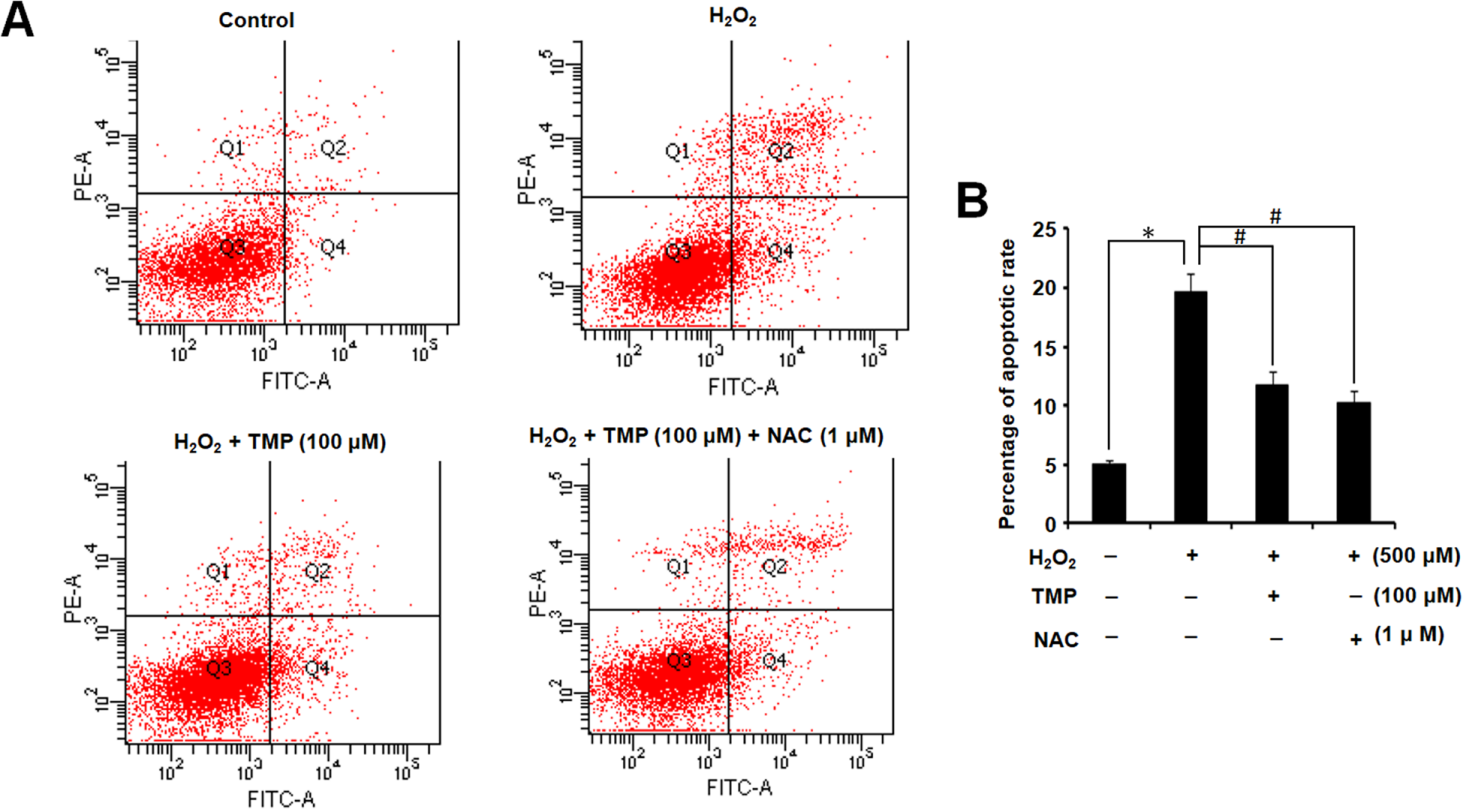
TMP alleviates the apoptosis of hUCMSCs. **a** hUCMSC apoptosis in each group was tested by flow cytometry. **b** Statistical analysis of the number of apoptosis. Columns, mean; bars, SEM. *P < 0.05 compared to the control group; ^#^*P* < 0.05 compared to the H_2_O_2_-treated group

The proportion of early apoptotic and late apoptotic hUCMSCs were counted as the total apoptosis rates. As presented in Fig. 4, compared with the control group, the apoptosis proportion of cells exposed to H_2_O_2_ (500 μM) was significantly increased. However, in pretreatment with TMP (100 μM) or NAC before exposure to H_2_O_2_, the percentage of apoptotic cells was crucially decreased, compared to the group treated with H_2_O_2_ only (*P* < 0.05).

### TMP activated Nrf2/HO-1 signaling pathway in H_2_O_2_-induced hUCMSCs

**Fig. 5:**
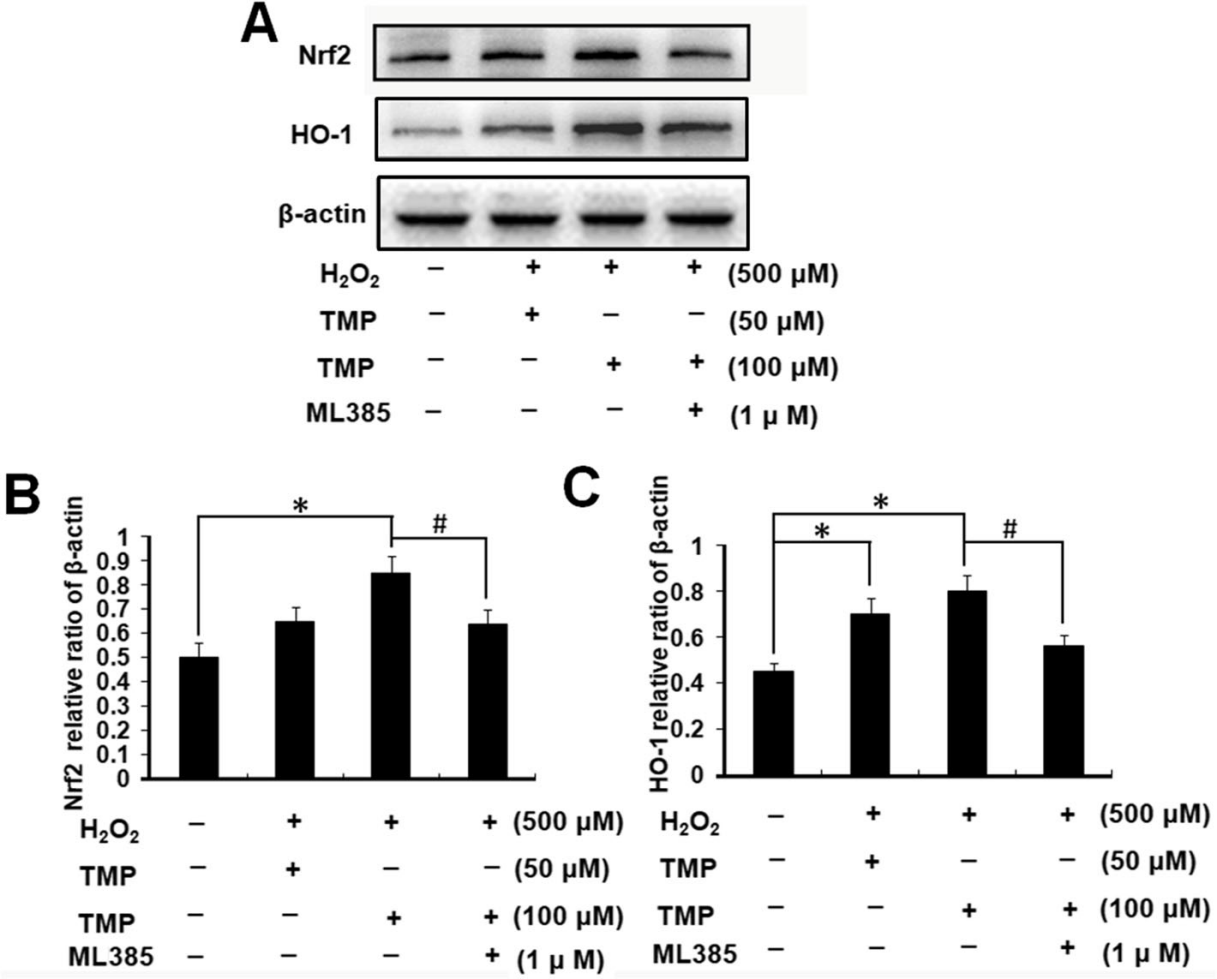
TMP protects hUCMSCs from oxidative injury by activating Nrf2/HO-1 pathway. **a** The expression of Nrf2 and HO-1 in each group. **b** Relative densitometric analysis of the protein bands. Columns, mean; bars, SEM. *P < 0.05 compared to the control group. The results are representative of three independent experiments

The expression of Nrf2 and HO-1 in each group was measured by western blot. We calculated the relative expressions of Nrf2 and HO-1. The results in Fig. 5 show that the expression level of Nrf2 in TMP (50, 100 μM) treated groups was remarkably higher than that in the control group (*P* < 0.05), and the upregulation of Nrf2 was significantly inhibited by ML385 (*P* < 0.05). The expression of HO-1 showed a similar expression pattern with Nrf2 that the expression level of HO-1 in TMP (50, 100 μM) significantly increased the expression of HO-1, compared to that of the control group (*P* < 0.05), and the upregulated expression was significantly decreased by ML385 (*P* < 0.05).

### TMP-hUCMSCs improved the clinical score of EAE mice

**Fig. 6:**
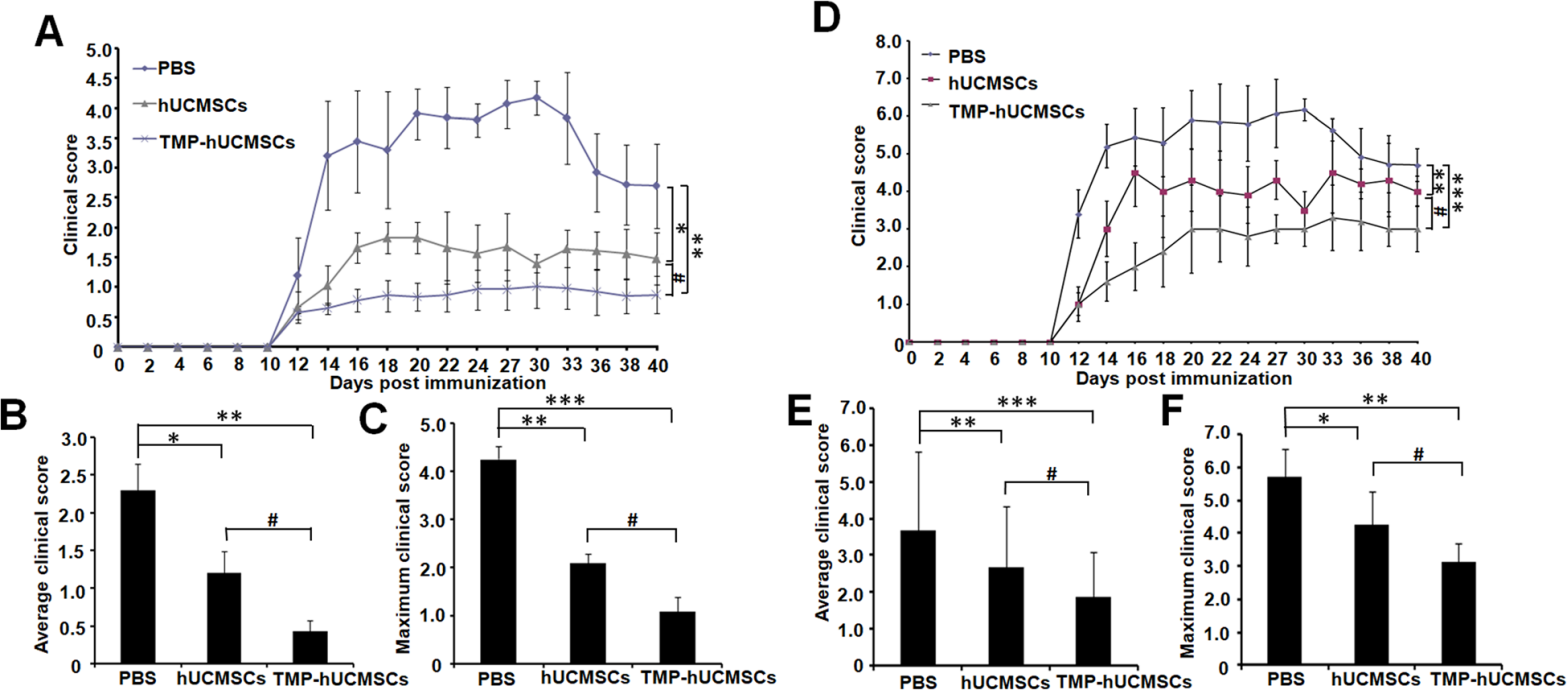
TMP-stimulated hUCMSCs attenuate clinical severity of EAE mice. **a** Mean daily clinical scores of each group recorded according to Kono’s 5 score (*n* = 20). **b** The average clinical score of each group recorded according to Kono’s 5 score. **c** The maximum clinical score of each mouse of each group recorded according to Kono’s 5 score. **d** Mean daily clinical scores of each group recorded according to Hooper’s 7 score (*n* = 20). **e** The average clinical score of each group recorded according to Hooper’s 7 score. **f** The maximum clinical score of each mouse of each group recorded according to Hooper’s 7 score. Columns, mean; bars, SEM. *P < 0.05, **P < 0.01, ***P < 0.001 compared to PBS treatment. ^#^*P* < 0.05. The results are representative of three independent experiments

To determine the effects of TMP-hUCMSC treatment in EAE mice, we administered PBS, hUCMSCs, and TMP-hUCMSCs to mice 13 days after immunization (*n* = 20/group). The EAE mice were scored daily until 22 days post-immunization according to the standard of Kono’s 5 score and Hooper’s 7 score. By analyzing the clinical scores recorded using those two methods, the results showed that hUCMSC and TMP-hUCMSC treatments both significantly decreased the average clinical score and the maximum clinical score, compared to PBS treatment (*P* < 0.05), and TMP-hUCMSC treatment significantly decreased the average clinical score and the maximum clinical score, compared to hUCMSC treatment (*P* < 0.05) (Fig. 6). These results indicate that TMP-hUCMSC treatment improved the neurological functional recovery of EAE mice.

### TMP-hUCMSC treatment reduced the inflammation of EAE mice

**Fig. 7:**
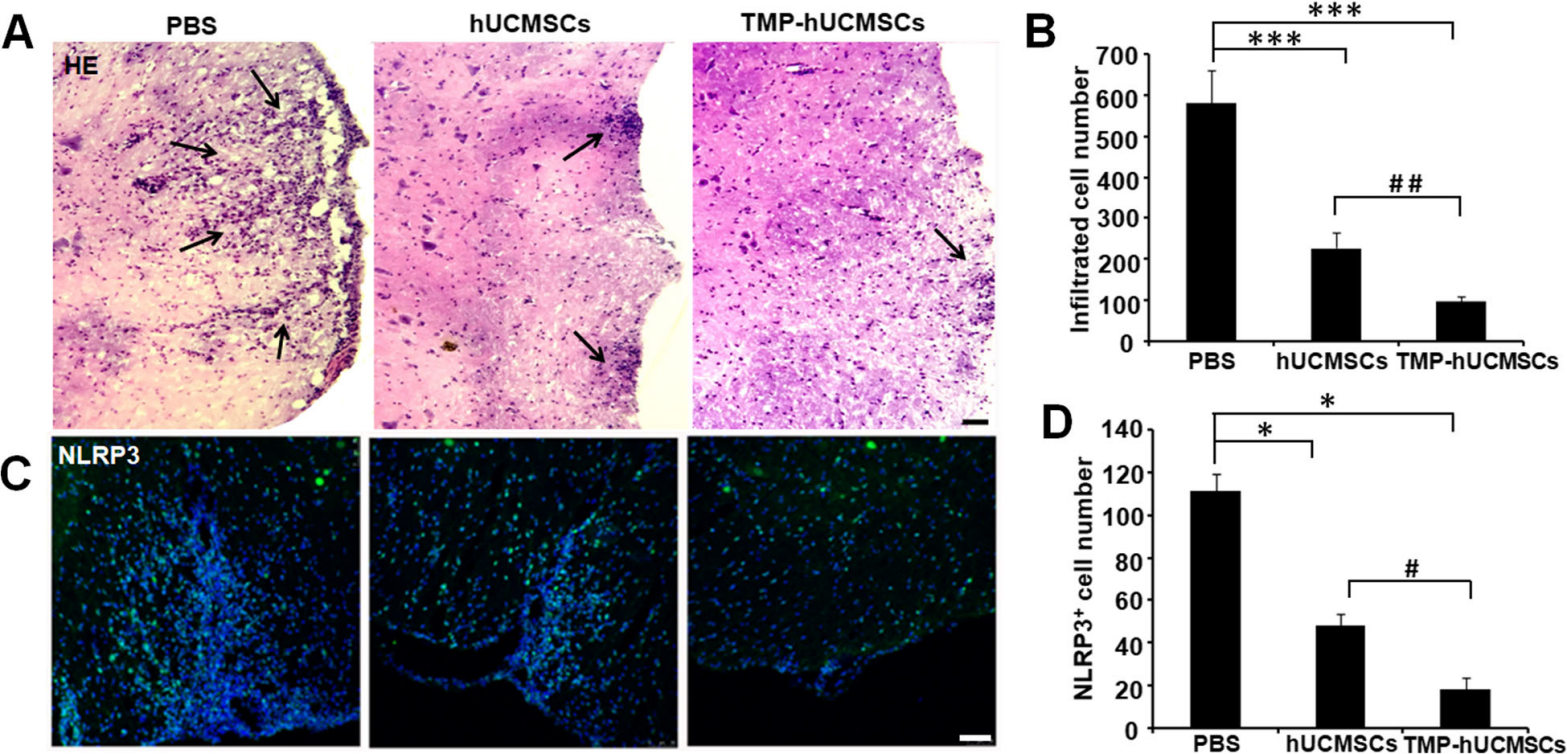
TMP-stimulated hUCMSCs decrease inflammation in EAE mice. **a** HE staining was performed to detect inflammatory cellular infiltration in each group. **b** Statistical analysis of the number of infiltrating cells. **c** NLRP3 staining was performed in each group. **d** Statistical analysis of the number of NLRP3-expressing cells in each mouse of each group. Columns, mean; scale bar = 50 μm, SEM. *P < 0.05, ***P < 0.001 compared to PBS treatment. ^#^*P* < 0.05, ^#*#*^*P* < 0.01 compared to hUCMSC treatment alone. The results are representative of three independent experiments

To detect the effects of TMP-hUCMSC treatment on inflammation in EAE mice, HE staining and immunofluorescence staining of NLRP3 were performed. HE results showed that hUCMSC and TMP-hUCMSC treatments both significantly decreased the inflammatory cellular infiltration, compared with PBS treatment (*P* < 0.001), and TMP-hUCMSC treatment more significantly decreased the inflammatory cellular infiltration, compared with hUCMSC treatment only (*P* < 0.01) (Fig. 7a, b). The activation of NLRP3 inflammasome aggravates the neuroinflammation and promotes the progression of EAE [20, 21]. Our results showed that hUCMSC and TMP-hUCMSC treatments both significantly decreased cell numbers of expressing NLRP3, compared with PBS treatment (*P* < 0.05), and the decrease in the TMP-hUCMSC-treated group was more significant, compared with the hUCMSC-treated group (*P* < 0.05) (Fig. 7c, d). The results suggest that TMP-hUCMSCs mitigated the inflammation to relieve the clinical severity of EAE.

### TMP-hUCMSC treatment decreased demyelination of EAE mice

**Fig. 8:**
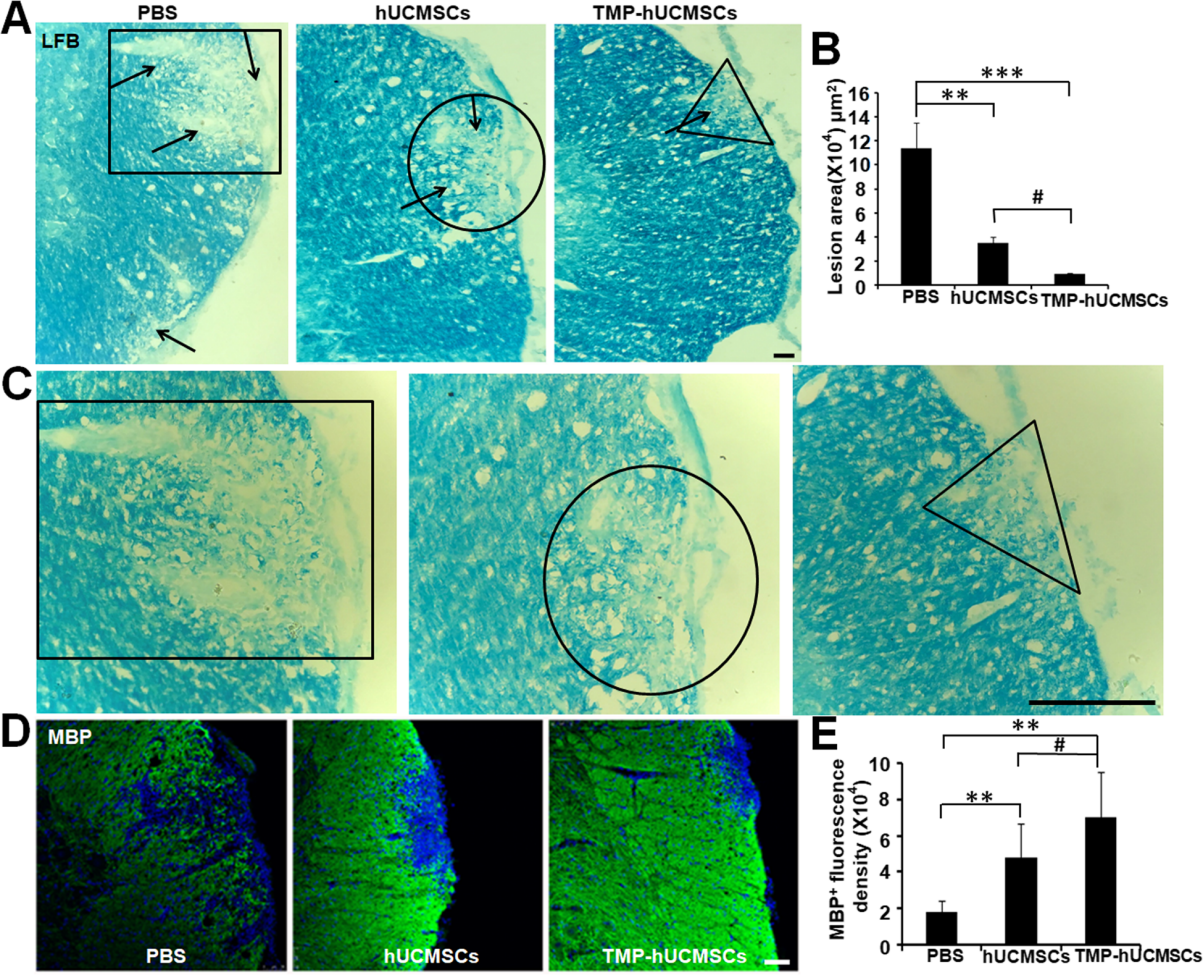
TMP-stimulated hUCMSCs decrease demyelination in EAE mice. **a** LFB staining was carried out to detect the extent of demyelination. The black arrowheads pointed to the demyelination area. **b** Statistical analysis of the demyelination in each group. **c** Detection of the extent of demyelination stained by LFB in a high magnification. The delineation of box, circle, and triangle in **c** is corresponding to those in **a**. **d** MBP staining in each group. **d** Statistical analysis of the fluorescence intensity of MBP in each mouse of each group. Columns, mean; scale bar = 50 μm, SEM. **P < 0.01, ***P < 0.001 compared to PBS treatment. ^#^*P* < 0.05 compared to hUCMSC treatment alone. The results are representative of three independent experiments

To determine the effects of TMP-hUCMSC treatment on the preservation of myelin of EAE mice, LFB staining and immunofluorescence staining of MBP were carried out. LFB staining showed that the extent of demyelination was significantly decreased in hUCMSC- or TMP-hUCMSC-treated mice, compared to PBS-treated mice (*P* < 0.01). Moreover, the significant reduction of demyelination was shown in TMP-hUCMSC-treated mice, compared to hUCMSC-treated mice (*P* < 0.05) (Fig. 8a–c). Furthermore, immunofluorescence staining of MBP showed that the preservation of myelin was significantly increased in the hUCMSC or TMP-hUCMSC treatment groups, compared to PBS treatment (*P* < 0.01), and the increasement in the TMP-hUCMSC-treated group was more significant, compared with the hUCMSC-treated group (*P* < 0.05) (Fig. 8d, e). These results suggest that TMP-hUCMSC treatment reduced demyelination to alleviate the clinical severity of EAE.

### TMP-hUCMSC treatment inhibited BBB disruption in EAE mice

**Fig. 9:**
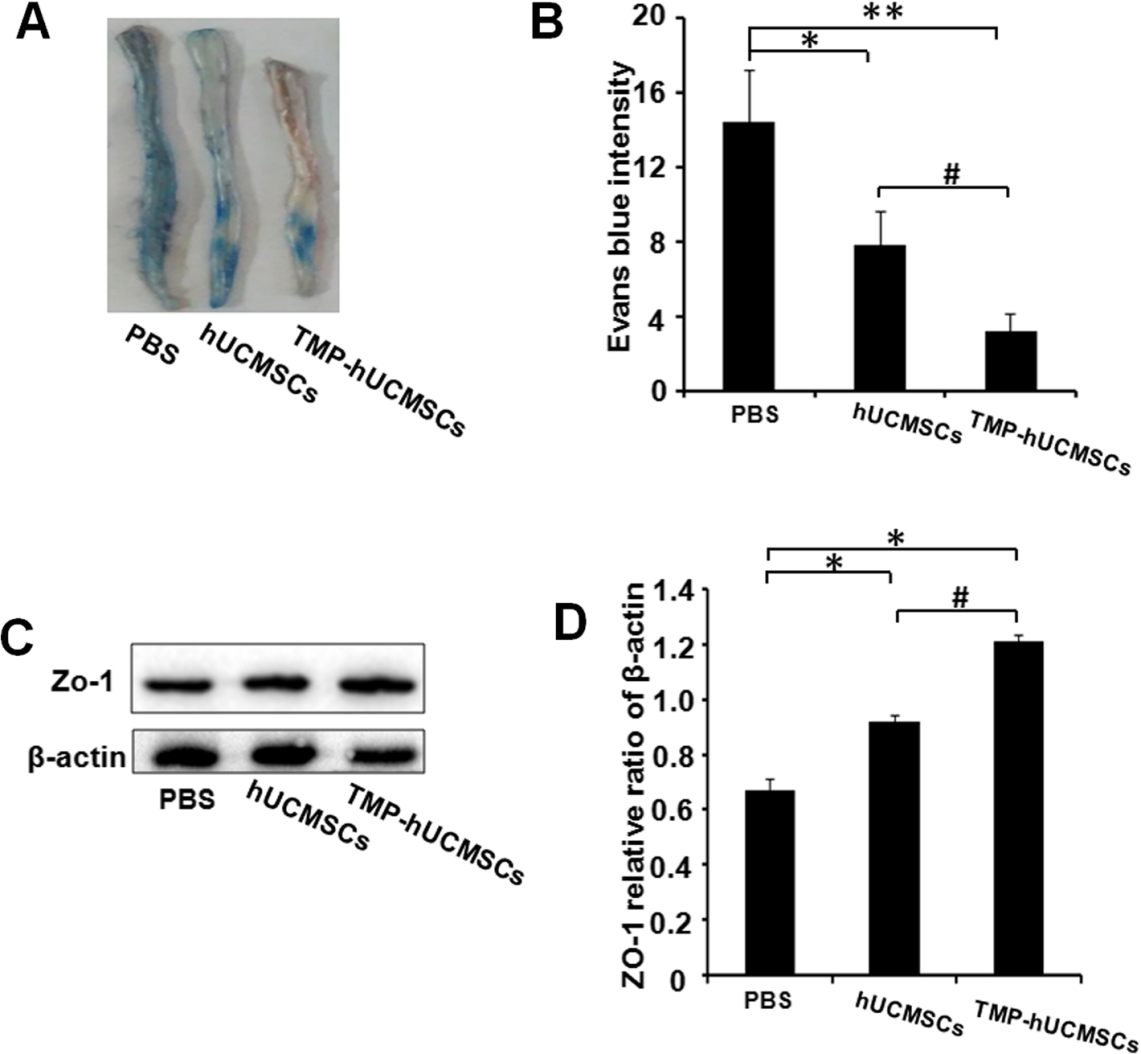
TMP-stimulated hUCMSCs inhibit BBB disruption in EAE mice. **a** Extravasation of EB dye in whole-mount spinal cords in each group. **b** Statistical analysis of the fluorescence intensity of EB in each group. **c** The expression of ZO-1 in the spinal cords of each group. **d** Relative densitometric analysis of the ZO-1 and β-actin. Columns, mean; bars, SEM. **P < 0.01, ***P < 0.001 compared to PBS treatment. ^#^*P* < 0.05 compared to hUCMSC treatment alone. The results are representative of three independent experiments

The extent of EB dye extravasation to the spinal cord was used to determine the effects of TMP-hUCMSCs on the protection of BBB integrity. The extent of EB dye extravasation showed a significant decrease in hUCMSC- or TMP-hUCMSC-treated mice, compared to PBS-treated mice (*P* < 0.05). Notably, TMP-hUCMSC treatment significantly reduced the extent of EB dye extravasation, compared to hUCMSC treatment (*P* < 0.05) (Fig. 9a, b). Moreover, the BSCB integrity was also assessed by analyzing the expression of ZO-1, which is a marker of tight junction protein [22]. As shown in Fig. 9c, d, the expression of ZO-1 was significantly elevated in mice treated with hUCMSCs, or TMP-hUCMSC treatment, compared to PBS treatment (*P* < 0.05). More importantly, TMP-hUCMSC treatment more significantly increased the expression of ZO-1, compared to hUCMSC treatment (*P* < 0.05). These results reveal that TMP-hUCMSC treatment protected the BBB integrity.

## Discussion

In the present study, we found that TMP (100 μM) alleviated hUCMSC apoptosis and promoted the hUCMSC proliferation by using an oxidative stress model in vitro. We also exhibited that TMP-hUCMSC treatment significantly reduced clinical scores, inflammation, demyelination, and BBB disruption in EAE mice compared with hUCMSC treatment alone.

hUCMSCs have been regarded as a potential therapeutic resource for clinical transplantation application for disease therapy due to their genetic stability, as well as functions in immunoregulation, stroma support, paracrine signaling, and migration [1]. However, the oxidative stress in local microenvironment is not conducive to their survival and inevitably leads to a large number of cell apoptosis during the process of transplantation therapy. Previous studies have reported that TMP has remarkable antioxidative and anti-apoptosis activities on human umbilical vein endothelial cells and bone marrow-derived MSCs against H_2_O_2_-induced apoptosis through PI3K/Akt and ERK1/2 pathways [9, 23]. In addition, MSCs pretreated with protective molecule enhance the therapeutic effects in EAE [24]. Consisting with these results, in our study, we showed that TMP changed the cell cycle that increased the number of hUCMSCs accumulated in the S phase and reduced the cell apoptosis induced by H_2_O2, which was used to mimic the oxidative stress in vitro. These may increase the number of viable cells in local microenvironment to amplify the therapeutic effects of hUCMSCs.

Abnormal cell cycle has been reported that was closely related to apoptosis [25]. Cell cycle arrest is one of the most important causes of apoptosis [26]. Accordingly, in this study, our results found that TMP changed the cell cycle that increased the number of hUCMSCs accumulated in the S phase and reduced the cell apoptosis induced by H_2_O_2_ [27, 28]. This indicates that TMP had protective effects on H_2_O_2_-induced hUCMSC apoptosis.

Oxidative stress is a result of overproduction of ROS, including a certain amount of superoxide anions, hydroxyl radicals, and hydrogen peroxide [29]. They can be produced by all vascular cells, smooth muscle cells, fibroblasts, adipocytes, and phagocytic cells in the transplant recipient [30]. Under normal physiological conditions, these cells produce an appropriate amount of ROS to play the role of phagocytosis and killing to protect the body against external or internal stressors. However, once the generation and clearance of ROS are out of balance, excessive ROS will lead to oxidative stress and ROS-mediated peroxidation damage of important organelles [31]. And then, apoptosis was triggered by cell death receptor and mitochondrial death pathway [23]. In this study, we induced the oxidative stress in vitro by treatment of hUCMSCs with H_2_O_2_. After treatment with H_2_O_2_, the ROS production was extremely huge. However, the ROS levels induced by H_2_O_2_ were significantly decreased by hUCMSCs or TMP pretreated hUCMSCs to inhibit oxidative stress and mitigate apoptosis. Moreover, TMP pretreated hUCMSCs more significantly decreased the ROS generation than hUCMSCs. The similar results were also shown in the expression of MDA, another marker for peroxidation, and opposite results of the expression of SOD, a marker for antioxidation. These results are consistent with previous study that inhibition of oxidative stress protects cell from apoptosis [32]. Taken together, TMP-hUCMSCs inhibited excessive ROS production to reduce the apoptosis.

In order to further explore the antioxidative potential mechanism of TMP, we detected the antioxidant stress-related pathway proteins, including Nrf2 and HO-1. Nrf2 is a key factor that is basically expressed in oxidative stress, and can be activated by the excessive production of ROS to initiate the transcription of HO-1. It has been demonstrated that inhibition of oxidative stress through the activation of Nrf2/HO-1 signaling pathway protects the H_2_O_2_-induced injury of intestinal epithelial cells and PC12 cells [33, 34]. In this study, the protein expression levels of Nrf2 and HO-1 were both significantly upregulated by TMP treatment. These results are in keeping with previous studies that TMP reduces oxidative stress in alcoholic liver by increasing the expression of Nrf2 [35], and TMP exhibits a neuroprotective effect by reducing apoptosis and oxidative stress through upregulated expression of Nrf2 and HO-1 [36]. Collectively, TMP may alleviate oxidative stress injury and cell apoptosis through activation of Nrf2/HO-1 pathway.

In conclusion, our study identified that TMP has protective effects on H_2_O_2_-induced hUCMSCs by activation of Nrf-2/HO-1 signaling pathway. Most importantly, TMP pretreatment amplified the therapeutic potential of hUCMSCs by preventing the cell apoptosis. Although further studies are required, such as detection of the apoptosis of the MSCs after transplantation, the therapeutic effects of MSCs administrated using different routes, and the neural-glial differentiation after transplantation [37], we can still propose that TMP has a promising clinical application in alleviating the apoptosis of hUCMSCs after transplantation into the recipient, and our study provides a new experimental suggestion for the treatment of numerous diseases, such as MS, stroke, brain trauma, and spinal cord injury.

## Data Availability

All data generated or analyzed during this study are included in this published article.

## Abbreviations

BBB: Blood-brain barrier
CCK-8: Cell count kit-8
CNS: Central nervous system
DCF: Dichlorofluorescein
DCFH-DA: 2,7-Dichlorofluorescein-diacetate
EAE: Experimental autoimmune encephalomyelitis
EB: Evans blue
HO-1: Heme oxygenase 1
HUCMSCs: Human umbilical cord mesenchymal stem cells
MDA: Malondialdehyde
NAC: *N*-Acetyl-l-cysteine
NLRP3: NOD-like receptor protein 3
Nrf2: Nuclear factor-erythroid 2-related factor-2
MBP: Myelin basic protein
MS: Multiple sclerosis
PI: Propidium iodide
ROS: Reactive oxygen species
SOD: Superoxide dismutase
TMP: Tetramethylpyrazine
